# Is Endurance Training Harmful to Older Athletes in the Long Run? An Interesting Case of Exercise-Induced Ventricular Tachycardia

**DOI:** 10.7759/cureus.19665

**Published:** 2021-11-17

**Authors:** Nader Lamaa, Jose L Batista, Amer Zeizoun, Kenneth Zide

**Affiliations:** 1 Cardiology, Aventura Hospital and Medical Center, Aventura, USA; 2 Internal Medicine, Aventura Hospital and Medical Center, Aventura, USA

**Keywords:** arvc, eps, exercise-induced vt, icd, right ventricle

## Abstract

There is limited data on how endurance training can impact cardiac function and arrhythmogenesis. Intense endurance training has been associated with pathological remodeling of the right ventricle (RV) that can act as a substrate for fatal ventricular arrhythmias in older athletes. A previously healthy 63-year-old female marathon runner presented with symptomatic monomorphic ventricular tachycardia (VT) while exercising. Transthoracic echocardiogram (TTE) demonstrated no structural or functional abnormalities. Electrophysiology study (EPS) with three-dimensional mapping and programmed electrical stimulation was performed demonstrating significant scarring of the RV, including RV outflow tract and RV free wall. VT ablation was successfully performed. Unfortunately, exclusion of arrhythmogenic right ventricular cardiomyopathy (ARVC) was limited due to the lack of cardiac magnetic resonance imaging (MRI). Therefore, a single chamber implantable cardioverter defibrillator (ICD) was placed for secondary prevention. Currently, the clinical significance of exercise-induced ventricular arrhythmias in trained athletes without cardiovascular disease is still unknown. This case highlights the need for investigation with larger studies and longer follow up to help us understand the mechanism of exercise-induced scar formation and standardize our management regarding screening, exercise recommendations, and ICD placement in older athletes.

## Introduction

There is limited data on how endurance training can impact cardiac function and arrhythmogenesis. Intense endurance exercise predominantly affects the right ventricle (RV) and may induce chronic structural changes and pathological remodeling that can act as a substrate for fatal ventricular arrhythmias [1]. Management and exercise recommendations remain a challenge as there is a deep gap across the literature. Here, we describe a case that entails the need for a standardized approach in diagnosing and managing older athletes with exercise-induced ventricular tachycardia (VT).

## Case presentation

**Figure 1:**
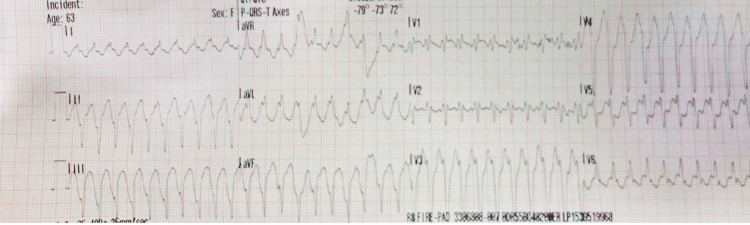
Initial electrocardiogram (EKG) at emergency medical service (EMS) arrival Wide complex tachycardia with a heart rate (HR) of 272 beats per minute corresponding to a tachycardia cycle length of 220 milliseconds.

**Figure 2:**
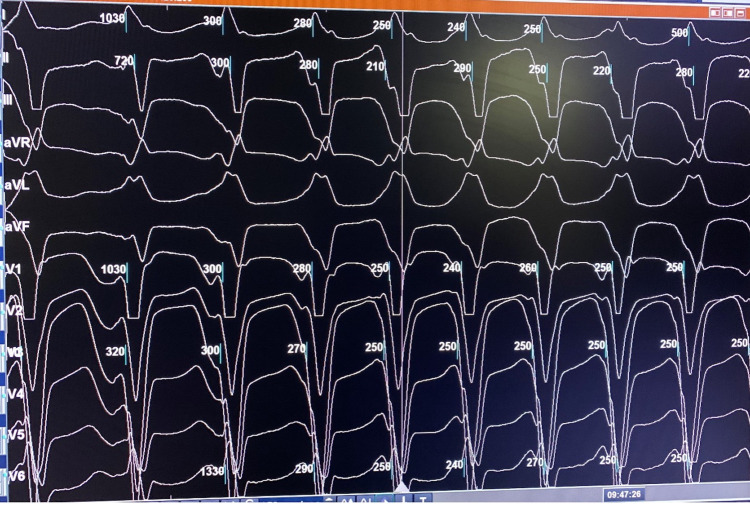
Electrophysiology study (EPS) Induced ventricular tachycardia (VT) showing same morphology as initial rhythm. Positive QRS axis in leads aVL, aVR, I, V6 and negative QRS axis in II, aVF, V1-V5.

**Figure 3:**
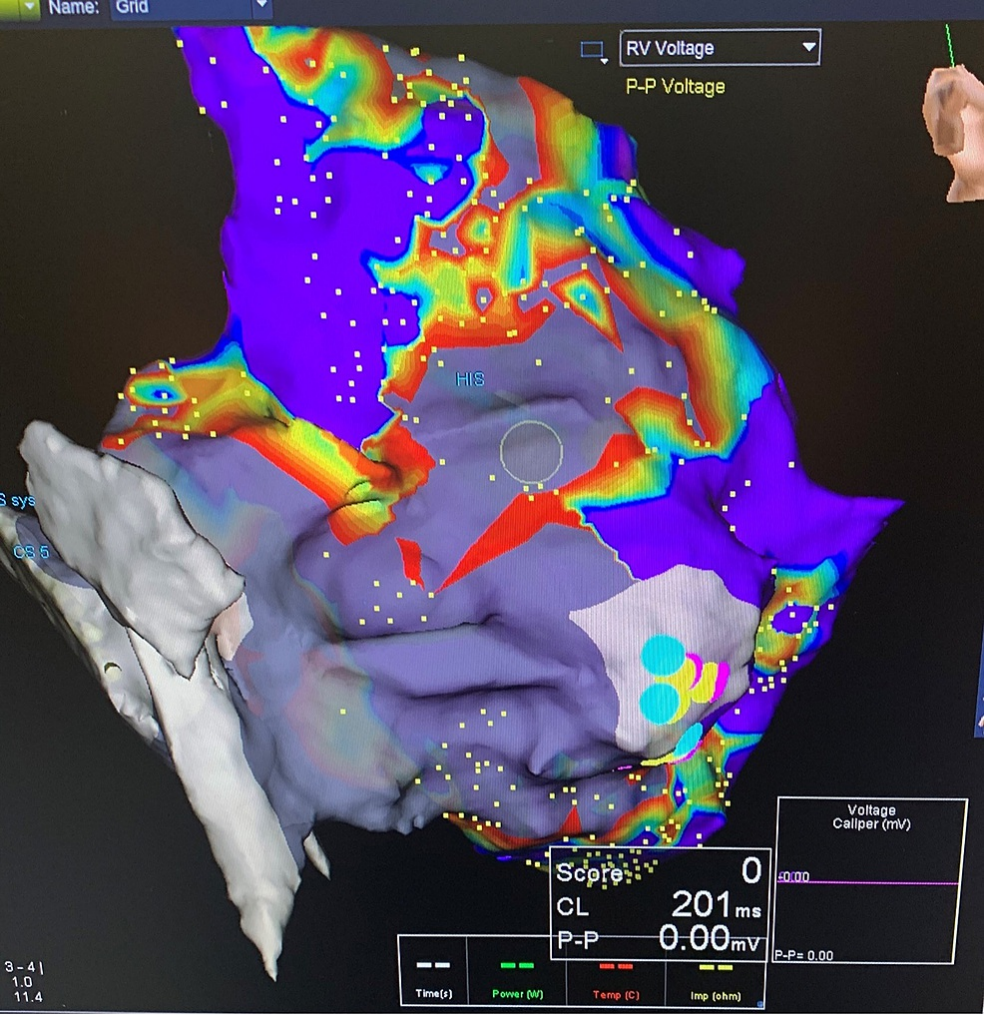
Three-dimensional (3D) anatomical map (EnSite*) Delineated scar <0.5 mV (red) serving as the substrate for ventricular tachycardia. *(EnSite Precision, Abbott, Saint Paul, MN, USA)

A previously healthy 63-year-old woman, who is an avid runner and still trains for and participates in marathons, presented with symptomatic wide complex tachycardia while exercising (Figure 1). Her symptoms were sudden onset - chest pain and palpitations, associated with a near syncope episode. Her vital signs were a blood pressure of 144/83 mmHg, oxygen saturation 100% on room air, heart rate of 74 bpm, and no presence of fever. A 100 Joules synchronized cardioversion administered by emergency medical services yielded a successful return to normal sinus rhythm, with a resolution of symptoms. Baseline electrocardiogram (EKG) showed normal sinus rhythm without any acute ischemic changes or pre-excitation. A transthoracic echocardiogram (TTE) demonstrated preserved ejection fraction (55%-60%) and coronary catheterization demonstrated no significant coronary artery disease. Further electrophysiology study (EPS) with three-dimensional (3D) mapping (EnSite Precision, Abbott, Saint Paul, MN, USA) and programmed electrical stimulation was performed (Figure 2). The EPS showed dual atrioventricular (AV) nodal physiology but no AV nodal echoes and no sustained VT. There was a significant scar seen by 3D mapping of the RV, especially concentrated in the right ventricular outflow tract (RVOT), inferior, and free wall (Figure 3). The induced VT (positive axis in aVL, aVR, I, V6 and negative axis in II, aVF, V1-V5) was similar to the presenting EKG, as seen in Figure 2. A hemodynamically unstable VT was ablated using an exact pace map at an area of scar in the inferior aspect of the RV at 25W and 40C with a 4-mm externally irrigated flexibility catheter (Abbott, Minneapolis, MN, USA). A single chamber implantable cardioverter defibrillator (ICD) was successfully placed for secondary prevention. The patient tolerated the procedure well without any peri procedural complications and was discharged the next day.

## Discussion

Our case showed an unusual substrate for VT localized into the RVOT, inferior and free wall in an older athlete without a prior history of underlying structural heart disease or cardiovascular risk factors. Currently, the clinical significance of exercise-induced ventricular arrhythmias in trained athletes without apparent cardiovascular disease remains unknown. In contrast to our case, in a large consecutive cohort of young and highly trained athletes, ventricular tachyarrhythmias induced by exercise testing were found in 7% [2]. The underlying mechanism of how an RV scar, similar to what we found in our patient, leads to a sudden episode of symptomatic VT remains unknown. Moreover, there is an ongoing debate on the expected normal physiologic changes of the right ventricle or ventricular function in athletes. In 2012, La Gerche et al. described a potential exercise-induced RV dysfunction and remodeling in endurance athletes where he suggested the term “load-induced, RV arrhythmogenic cardiomyopathy‟, which could stem from repetitive microtraumas to chronic and structural changes in RV and pro-arrhythmogenesis [1]. In his view, VT originating from the RV is responsible for acute deaths, a hypothesis based on a retrospective analysis of electrophysiological examinations. On the other hand, distinguishing exercise-induced VT from arrhythmogenic right ventricular cardiomyopathy (ARVC) is crucial and has important clinical and prognostic implications. It is not clear, however, that the same risk factors for younger athletes are applicable to athletes over the age of 60 years old. A thorough history, genetic testing, cardiac imaging including cardiac magnetic resonance imaging (MRI) and RV strain, and electroanatomic mapping can all aid in making that distinction [3]. Unfortunately, our diagnosis was limited due to the lack of cardiac MRI at our institution. Therefore, the decision was made to implant an ICD for secondary prevention since we were not able to exclude the possibility of VT recurrence. 

Furthermore, this case suggests that EPS and 3D mapping can be important to guide us to the possible underlying mechanism and formulate further treatment plans for similar patients. This was illustrated by a recent study that described a novel clinical entity of an isolated subepicardial RVOT scar as a substrate for fast VT in high-level endurance athletes that can be successfully treated by ablation [4]. Of interest, in their paper, they report that the specific scar pattern was identified by electroanatomic mapping but not by imaging (cardiac MRI). 

## Conclusions

Our observations posit a few important questions. Is endurance training the only exposure to form VT substrate or should we consider genetic testing in all these patients? What is the optimal method to screen asymptomatic older athletes who wish to continue high-intensity exercise? Is there any benefit to defibrillator therapy in this population? What are the best recommendations for symptomatic older athletes post ablation therapy? There is not enough data in the literature to answer these questions. This case highlights the need for investigation with larger studies and longer follow up to help us understand the mechanism of exercise-induced scar formation and standardize our management regarding screening, exercise recommendations, and ICD placement in older athletes.
